# Supplementary material for: Muscle Sonography in Inclusion Body Myositis: A Systematic Review and Meta-Analysis of 944 Measurements
Source: Cells. 2022 Feb 9;11(4):600. doi: 10.3390/cells11040600 (PMC8869828; doi:10.3390/cells11040600)
Supplement: Supplementary file 1 [file cells-11-00600-s001.zip › cells-1542491-supplementary.pdf]

Table S1. Meta-regression for US device.

Variable	Coefficient	95% CI	P value
**FDP echogenecity**			
Esaote Mylab Twice	1		
GE LOGIQ e	11.47	(2.99 – 19.94)	0.01
Ge LOGIQ 7	20.21	(1.90 – 38.52)	0.03
**Gastrocnemius echogenecity**			
Esaote Mylab Twice	1		
GE LOGIQ e	6.00	(-19.41 – 31.40)	0.64
Ge LOGIQ 7	33.67	(0.17 – 67.17)	0.05

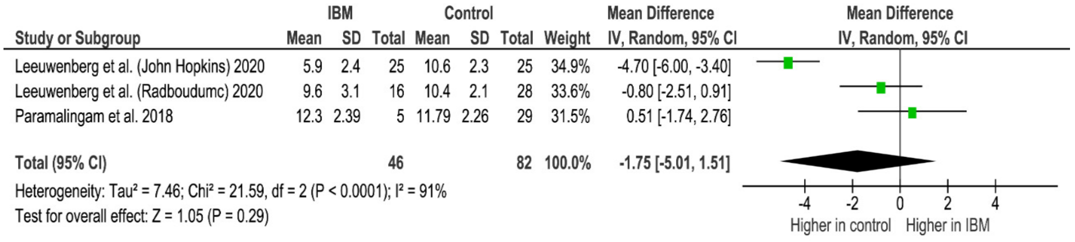

Figure S1. Forest plot of the muscle thickness of FDP in mm of the included studies.

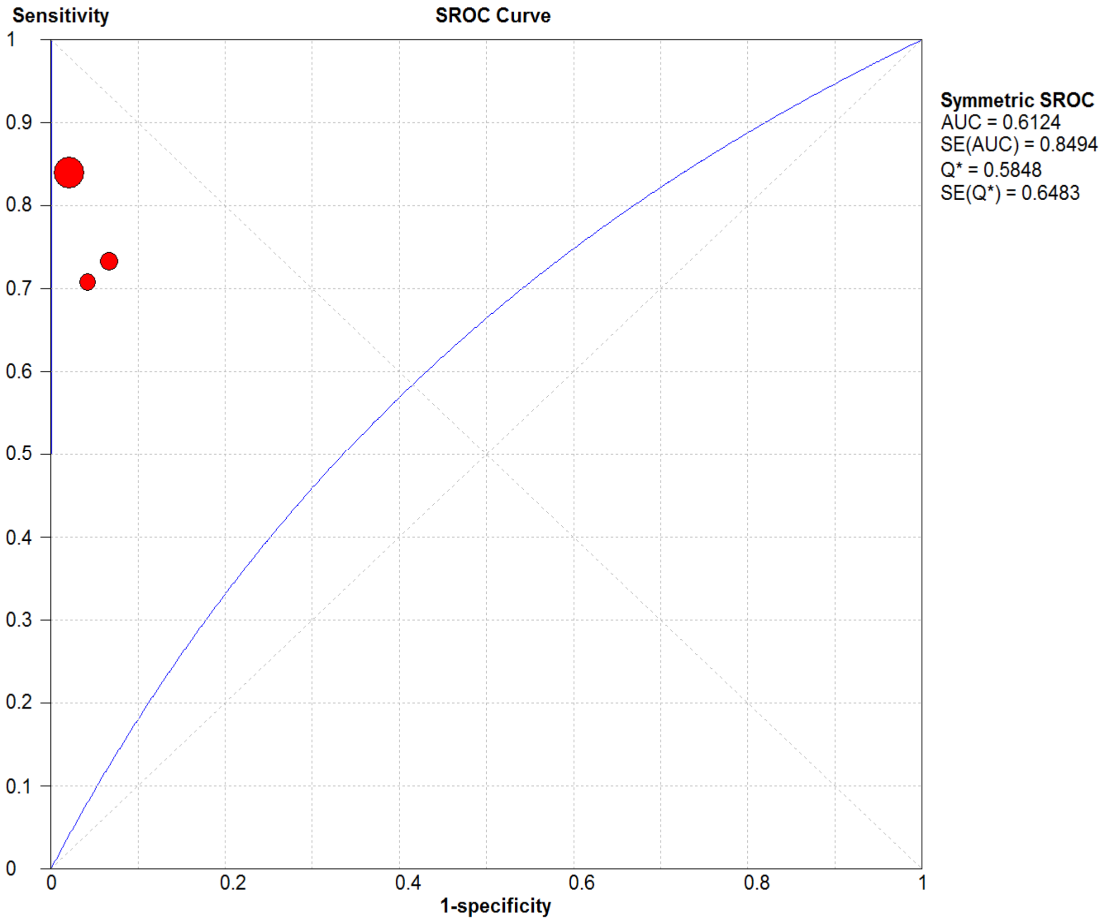

Figure S2. SROC curve shows the AUC was 0.612 and the value with the highest sensitivity and specificity (Q* index) was 0.5848.

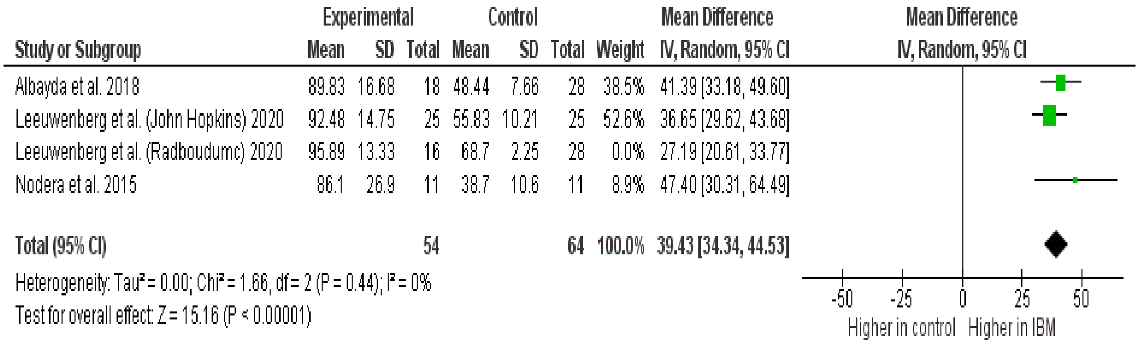

Figure S3. Sensitivity analysis for FDP echogenicity meta-analysis.

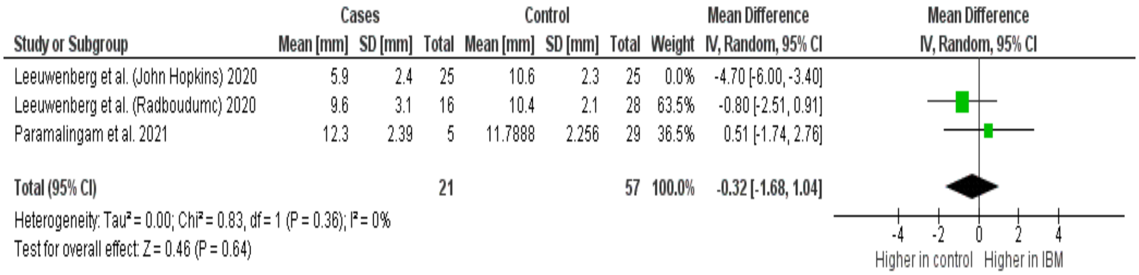

Figure S4. Sensitivity analysis for muscle thickness meta-analysis.

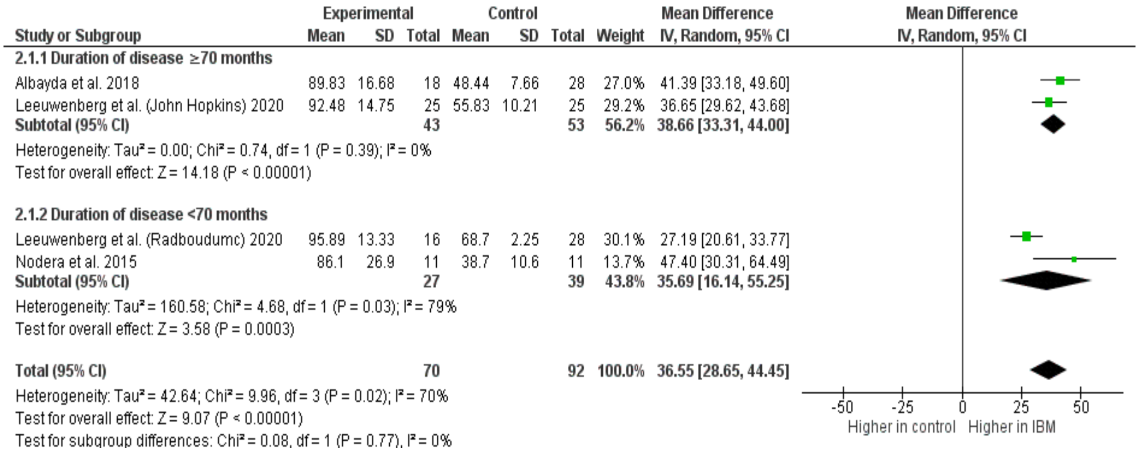

Figure S5. Subgroup analysis for FDP echogenicity meta-analysis by disease duration.

Study or Subgroup	Experimental Mean	Experimental SD	Experimental Total	Control Mean	Control SD	Control Total	Weight	Mean Difference IV, Random, 95% CI
**2.2.1 Duration of disease ≥70 months**								
Albayda et al. 2018	91.41	13.55	18	59.49	12.02	28	27.7%	31.92 [24.24, 39.60]
Leeuwenberg et al. (John Hopkins) 2020	91.18	12.43	25	73.86	12.71	25	28.3%	17.32 [10.35, 24.29]
**Subtotal (95% CI)**			**43**			**53**	**55.9%**	**24.53 [10.22, 38.83]**
Heterogeneity: Tau² = 92.58; Chi² = 7.61, df = 1 (P = 0.006); I² = 87%								
Test for overall effect: Z = 3.36 (P = 0.0008)								
**2.2.3 Duration of disease <70 months**								
Leeuwenberg et al. (Radboudumc) 2020	92.78	17.97	16	74.25	8.23	34	26.2%	18.53 [9.30, 27.76]
Nodera et al. 2015	103.2	30.4	11	51	2.9	11	17.8%	52.20 [34.15, 70.25]
**Subtotal (95% CI)**			**27**			**45**	**44.1%**	**34.44 [1.49, 67.38]**
Heterogeneity: Tau² = 513.36; Chi² = 10.60, df = 1 (P = 0.001); I² = 91%								
Test for overall effect: Z = 2.05 (P = 0.04)								
**Total (95% CI)**			**70**			**98**	**100.0%**	**27.90 [16.32, 39.48]**
Heterogeneity: Tau² = 110.91; Chi² = 18.32, df = 3 (P = 0.0004); I² = 84%								
Test for overall effect: Z = 4.72 (P < 0.00001)								
Test for subgroup differences: Chi² = 0.29, df = 1 (P = 0.59), I² = 0%								

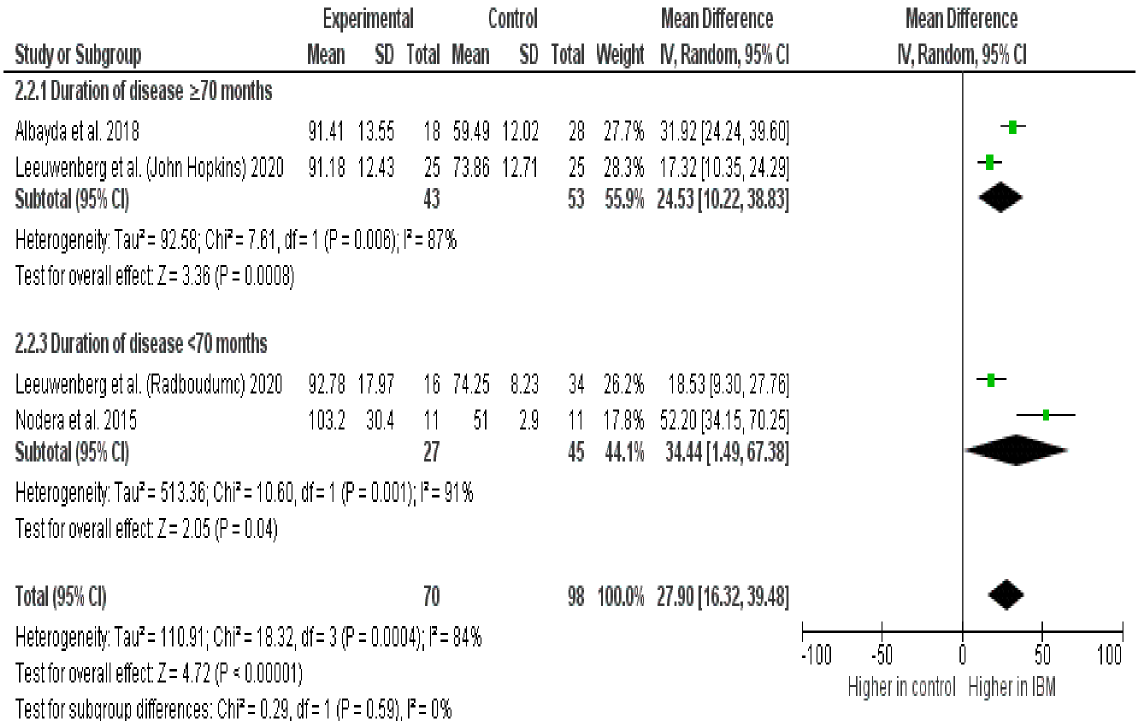

Figure S6. Subgroup analysis for GC echogenicity meta-analysis by disease duration.
